# Betulinic Acid Induces ROS-Dependent Apoptosis and S-Phase Arrest by Inhibiting the NF-*κ*B Pathway in Human Multiple Myeloma

**DOI:** 10.1155/2019/5083158

**Published:** 2019-06-09

**Authors:** Min Shen, Yiqiang Hu, Yan Yang, Lanlan Wang, Xin Yang, Bo Wang, Mei Huang

**Affiliations:** ^1^Department of Hematology, Tongji Hospital, Tongji Medical College, Huazhong University of Science and Technology, Wuhan 430030, China; ^2^Department of Orthopaedics, Union Hospital, Tongji Medical College, Huazhong University of Science and Technology, Wuhan 430022, China; ^3^Department of Hematology, Wuhan First Hospital, Huazhong University of Science and Technology, Wuhan 430022, China; ^4^Department of Rehabilitation, Union Hospital, Tongji Medical College, Huazhong University of Science and Technology, Wuhan 430022, China

## Abstract

Betulinic acid (BA), as a prospective natural compound, shows outstanding antitumor bioactivities against many solid malignancies. However, its mechanism against multiple myeloma (MM) remains elusive. Herein, for the first time, we studied the antitumor activity of BA against MM both *in vivo* and *in vitro*. We showed that BA mediated cytotoxicity in MM cells through apoptosis, S-phase arrest, mitochondrial membrane potential (MMP) collapse, and overwhelming reactive oxygen species (ROS) accumulation. Moreover, when the ROS scavenger N-acetyl cysteine (NAC) effectively abated elevated ROS, the BA-induced apoptosis was partially reversed. Our results revealed that BA-mediated ROS overproduction played a pivotal role in anticancer activity. Molecularly, we found that BA resulted in marked inhibition of the aberrantly activated NF-*κ*B pathway in MM. As demonstrated by using the NF-*κ*B pathway-specific activator TNF-*α* and the inhibitor BAY 11-7082, BA-mediated inhibition of the NF-*κ*B pathway directly promoted the overproduction of ROS and, ultimately, cell death. Furthermore, BA also exerted enormous tumor-inhibitory effects via repressing proliferation and inhibiting the NF-*κ*B pathway in our xenograft model. Overall, by blocking the NF-*κ*B pathway that breaks redox homeostasis, BA, as a potent NF-*κ*B inhibitor, is a promising therapeutic alternative for MM.

## 1. Introduction

Multiple myeloma (MM), an incurable plasma cell cancer, is the second most prevalent hematological malignancy [1]. Many patients do not respond to current therapies and ultimately succumb to the disease, primarily because of drug resistance, apoptosis evasion, and an ability to grow independent of the bone marrow microenvironment, especially for patients in high-risk biological states [2, 3]. Thus, there is an urgent need to find effective drugs for treating MM.

Betulinic acid (BA), a lupane-structured pentacyclic triterpenoid, is a plant-derived product ubiquitously distributed throughout the plant kingdom [4]. Previous studies have demonstrated its multiple bioactivities, such as antitumor, anti-inflammatory, anti-HIV, and hepatoprotective activities [5, 6]. Among these properties, the antitumor activities have attracted considerable attention worldwide. Indeed, without causing obvious toxicity toward normal cells [7], growing evidence indicates that BA exhibits significant cytotoxicity against many malignancies [4, 8–10]. However, with regard to MM, its underlying mechanisms and relevant molecular targets have not yet been thoroughly elucidated.

Reactive oxygen species (ROS), which are byproducts of normal mitochondrial metabolism and homeostasis, include oxygen free radicals and nonradical oxidants such as O_2_^−^, OH^−^, H_2_O_2_, and NO [11, 12]. ROS generally play a dual role in living systems [13]. Specifically, ROS play a beneficial role at low or moderate levels, whereas a burst of ROS can destroy cellular homeostasis, as manifested by disruption of the mitochondrial membrane potential (MMP), release of cytochrome c (cyt c), activation of the caspase cascade, and ultimate induction of apoptosis [11, 14]. BA-induced cell death via excessive ROS has actually been reported in other tumors [15, 16], and we hypothesized that BA may act in a similar way against MM.

The nuclear factor-kappa B (NF-*κ*B) pathway, a key link between inflammation and cancer, plays important roles in inflammation, cell proliferation, and apoptosis [17–20]. Aberrant and stable activation of NF-*κ*B signaling has been reported in a wide range of malignancies, particularly MM [18, 21, 22]. Although the BA-mediated transcriptional activities of NF-*κ*B vary in cancers [10, 23], the dysregulation of the NF-*κ*B pathway is closely related to ROS levels and cellular redox balance [24–26]; however, this phenomenon has not yet been tested in MM, and its potential mechanism remains a mystery.

Accordingly, we aimed to evaluate the efficacy of BA against MM both *in vitro* and *in vivo*, to investigate the underlying mechanisms, and to illuminate the complex regulatory interactions between ROS and the NF-*κ*B pathway.

## 2. Material and Methods

### 2.1. Reagents and Antibodies

BA (purity ≥ 98%, Sigma-Aldrich, St. Louis, MO, USA) was dissolved in DMSO (Sigma-Aldrich) as a 40 mM stock solution and stored at −20°C. BAY 11-7082 and N-acetyl cysteine (NAC) were purchased from Beyotime (Shanghai, China). TNF-*α* was acquired from PeproTech (Rocky Hill, NJ, USA). Primary antibodies specific for Bax, Bcl-2, cleaved caspase-3, NF-*κ*B p65, NF-*κ*B p65 (phosphoS536), I*κ*B*α*, I*κ*B*α* (phosphoS32), cleaved PARP1, cyt c, CDK2, and cyclin A2 were purchased from Abcam (Cambridge, MA, USA). Primary antibodies specific for cleaved caspase-8, cleaved caspase-9, phospho-IKK*α*/*β* (Ser176/180), GAPDH, p21^Waf1/Cip1^, and p27^Kip^ were purchased from Cell Signaling Technology (Danvers, MA, USA). Secondary rabbit anti-mouse and goat anti-rabbit antibodies were purchased from Santa Cruz Biotechnology (Dallas, Texas, USA).

### 2.2. Cell Culture and Treatments

Human multiple myeloma U266 and RPMI 8226 cell lines were obtained from the China Center for Type Culture Collection (CCTCC). U266 and RPMI 8226 cells were routinely cultured in RPMI 1640 medium (HyClone, Logan, UT, USA) with 10% fetal bovine serum (FBS, Thermo Fisher Scientific, Waltham, MA, USA) and 1% penicillin/streptomycin (Beyotime, China). The cells were maintained in a humidified incubator at 37°C with 5% CO_2_ and subcultured at approximately 80-90% confluence. The cells were treated with various concentrations of BA (10, 20, and 40 *μ*M), and DMSO (0.1%) was used as a vehicle/control.

### 2.3. Hoechst 33342 Staining

Apoptotic cells were detected by Hoechst 33342 (Invitrogen, Carlsbad, CA, USA) staining. BA-treated cells were collected and fixed with 1 ml of 4% paraformaldehyde for approximately 20 min. After fixation, the cells were washed three times with PBS and incubated with 1 ml of Hoechst 33342 dye for 20 min in the dark. Morphological changes in nuclear chromatin were observed under a fluorescence microscope (Olympus, Japan).

### 2.4. CCK-8 and EdU Proliferation Assay

Cell viability was estimated using the CCK-8 kit (Dojindo, Kumamoto, Japan). MM cells were seeded in 100 *μ*l of medium in 96-well plates at a density of 1 × 10^4^ cells per well, and 10 *μ*l of CCK-8 solution was added after 6, 12, 24, and 48 h of incubation. The absorbance at 450 nm was measured 4 h later. Each experiment was performed independently in triplicate. Cell proliferation was detected using the Click-iT™ EdU-555 kit (Life Technologies, Grand Island, NY, USA) according to the manufacturer's instructions. After staining, fluorescence images were obtained using a fluorescence microscope (Olympus, Japan).

### 2.5. Annexin V/Propidium Iodide (PI) Double Staining

Apoptosis was detected by annexin V/PI (BD Biosciences, San Jose, CA, USA) double staining. The cells were harvested and washed twice with PBS at 4°C, resuspended in 200 *μ*l of binding buffer, and labeled with 5 *μ*l of annexin V and 5 *μ*l of PI in the dark for 15 min. The total apoptosis ratio was calculated as the sum of early and late apoptosis, as detected by flow cytometry (BD LSR II, USA).

### 2.6. Cell Cycle Analysis

The cell cycle was analyzed using a cell cycle kit (BD Biosciences) according to the manufacturer's protocol. Briefly, the cells were collected and fixed overnight in 70% ethanol at −20°C. After centrifugation at 600 ×g for 5 min, the cells were rehydrated in 3 ml of PBS for 15 min and then resuspended and incubated in 1 ml of DNA staining solution at 37°C for 30 min in the dark. The cell cycle distribution was detected by flow cytometry.

### 2.7. Measurement of MMP (Δ*ψ*M)

A JC-1 assay kit (Beyotime) was used to explore MMP transition. The cells were harvested, loaded with a 10 *μ*M concentration of the JC-1 probe, and then incubated at 37°C with 5% CO_2_ for 20 min in the dark. After incubation, the cells were washed twice with cold staining buffer and resuspended. The MMP transition was measured by flow cytometry, and fluorescence graphs were acquired using a fluorescence microscope (Olympus, Japan).

### 2.8. Evaluation of ROS

Intracellular ROS generation was measured using a ROS detection kit (Invitrogen). The cells were stained with a 2,7-dichlorofluorescin diacetate (DCFH-DA) probe (10 *μ*M) at 37°C for approximately 30 min and washed with serum-free medium. The median fluorescence intensity of ROS was measured by flow cytometry. All steps were strictly executed according to the manufacturer's instructions.

### 2.9. Malondialdehyde (MDA) Assay and Superoxide Dismutase (SOD) Assay

Lipid peroxidation was evaluated by a MDA assay kit (Nanjing Jiancheng Bioengineering Institute, China) based on the thiobarbituric acid (TBA) method. The absorbance was measured at 532 nm. Intracellular antioxidant SOD activity was assessed using a commercially available kit (Nanjing Jiancheng Bioengineering Institute) based on the autooxidation of hydroxylamine. The absorbance was measured at 550 nm. All the procedures were carried out following the manufacturer's instructions.

### 2.10. Real-Time PCR

After 12 hours of treatment with 40 *μ*M BA, total RNA was extracted from U266 cells using the TRIzol reagent (Invitrogen) according to the manufacturer's protocol. The isolated RNA was subsequently reverse-transcribed into complementary DNA with reverse transcriptase (Toyobo, Japan). The PCR reaction mixture was prepared using an SYBR Green master mix (Toyobo) with primers as follows: GAPDH: forward 5 ′-AATCCCATCACCATCTTCCAG-3 ′ and reverse 5 ′-GAGCCCCAGCCTTCTCCAT-3 ′; SOD2: forward 5 ′-AACCTCACATCAACGCGCA-3 ′ and reverse 5 ′-TCTCCTCGGTGACGTTCAGG-3 ′; FHC: forward 5 ′-CATCAACCGCCAGATCAACC-3 ′ and reverse 5 ′-CACATCATCGCGGTCAAAGT-3 ′; GCLM: forward 5 ′-ACCTCTGATCTAGACAAAACACAGT-3 ′ and reverse 5 ′-ACACAGCAGGAGGCAAGATTA-3 ′; and GSTM: forward 5 ′-AACCAGTTTATGGACAGCCG-3 ′ and reverse 5 ′-AGGCAGCTGGGCTCAAATAC-3 ′. The real-time quantitative PCR was performed using an ABI 7900HT Sequence Detection System (Applied Biosystems, Foster City, CA). Relative quantification of target mRNA expression was calculated using the 2^(−ΔΔCT)^ method and further normalized to GAPDH mRNA.

### 2.11. Western Blot Analysis

RIPA lysis buffer (Sigma) containing a proteinase inhibitor cocktail (Servicebio, Wuhan, China) and phenylmethanesulfonyl fluoride (Servicebio) was used to extract total proteins. The protein concentration was measured using a BCA assay (Invitrogen). Proteins (30 *μ*g) were separated by 12% SDS-PAGE and electrotransferred onto polyvinylidene fluoride (PVDF) membranes (Millipore, USA). The membranes were blocked with 5% nonfat milk for 1 h, followed by overnight incubation with specific primary antibodies at 4°C. The membranes were washed three times for 10 min each, incubated with a secondary antibody for 1 h at room temperature, and washed again. Finally, the bands were visualized using an ECL kit (Thermo Scientific, Rockford, IL, USA).

### 2.12. Immunofluorescence Staining

Cells were attached to glass slides using cytospin, fixed with 4% paraformaldehyde, and permeabilized with 0.3% Triton X-100. After being blocked with 5% BSA for 15 min at room temperature, the cells were incubated overnight at 4°C with primary antibody, washed 3 times with PBS, and stained with an Alexa Fluor 488-conjugated secondary antibody for 1 h. Nuclei were stained with Hoechst 33342. Images were captured using a fluorescence microscope. The standard semiquantitative evaluation of NF-*κ*B p65 was performed using ImageJ software (NIH, Bethesda, MD).

### 2.13. Nude Mouse Xenograft Model

Experiments involving animals were approved by the Experimental Animal Ethical Committee of Huazhong University of Science and Technology and were conducted in strict accordance with the National Institutes of Health Guide for the Care and Use of Laboratory Animals. Five-week-old BALB/c nude mice (16-20 g) were purchased from Beijing HFK Bioscience. U266 cells (1 × 10^7^) were suspended in 100 *μ*l of PBS and subcutaneously inoculated into the axilla of the right forelimbs of the mice. Nine days later, the majority of tumors grew to approximately 100 mm^3^, and the tumor-bearing mice were randomly assigned into two groups (five mice per group). The control and experimental groups were intraperitoneally administered a solvent (100 *μ*l of PBS with 0.1% DMSO) or BA (20 mg/kg), respectively, at 3-day intervals. Using a caliper, the tumor volume was calculated as the length (mm) × width (mm)^2^/2. At the end of the experiment, the mice were sacrificed, and the tumors were isolated for further study.

### 2.14. Immunohistochemistry (IHC)

Subcutaneous xenograft tumors were prepared for IHC analysis. IHC was performed in a Leica Bond Max automated system (Leica Biosystems, Nussloch, Germany) using the Leica-Refine detection kit (Leica Biosystems, DS9800). Briefly, after deparaffinization, rehydration, and antigen retrieval, the sections were incubated with an anti-Ki-67 antibody (1 : 200, Abcam) and an anti-NF-*κ*B p65 antibody (1 : 100, Abcam) for 30 min at room temperature. After washing, the sections were incubated with a horseradish peroxidase-conjugated secondary antibody, visualized with diaminobenzidine, and counterstained with hematoxylin. The stained slides were observed under a microscope (Olympus, Japan).

### 2.15. Statistical Analysis

Data obtained from at least three separate experiments are presented as the means ± SD. Differences between three or more groups were assessed using one-way ANOVA, followed by Tukey's multiple comparison test. Two-tailed Student's *t*-test was used in the analysis of two-group parameters. All statistical analyses were performed using GraphPad Prism 6.0. *P* < 0.05 was defined as statistically significant.

## 3. Results

### 3.1. BA Promotes Morphological Changes in MM Cells

**Figure 1:**
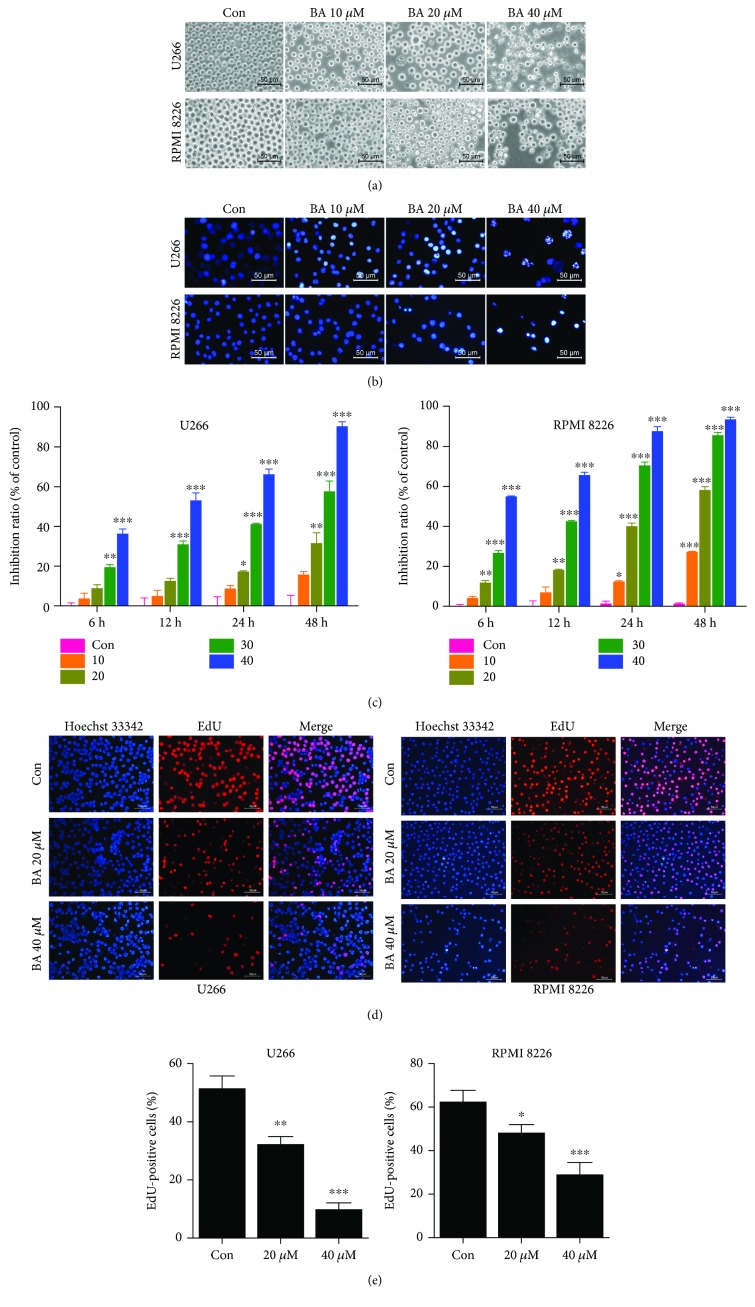
BA changed the morphology and inhibited the proliferation of MM cells. U266 and RPMI 8266 cells were exposed to different concentrations of BA (10, 20, 30, and 40 *μ*M) or 0.1% DMSO (control group) for 12 h. (a) BA induced obvious morphological changes, with shrunken and broken dead cells visible under phase-contrast microscopy. (b) Apoptotic cells with nuclear pyknosis and asymmetric chromatin condensation were brightly stained with Hoechst 33342. (c) CCK-8 was used to evaluate inhibitory effects after treatment with different concentrations of BA for various hours (6, 12, 24, and 48 h). The results are presented as the mean ± SD of three independent experiments. ^∗^*P* < 0.05; ^∗∗^*P* < 0.01; and ^∗∗∗^*P* < 0.001. (d) EdU staining was used to detect cell proliferation. EdU-positive cells (red fluorescence) were significantly decreased in a concentration-dependent manner after BA treatment for 12 h. (e) Quantitative analysis of EdU-positive cells. ^∗^*P* < 0.05; ^∗∗^*P* < 0.01; and ^∗∗∗^*P* < 0.001.

Incubation of MM cells with different concentrations of BA for 12 h elicited marked morphological changes that included shrunken and broken dead cells and cell debris under phase-contrast microscopy (Figure 1(a)). Apoptotic cells with wrinkled membranes, condensed nuclei, and fragmented chromatin were brightly stained and clearly visible after Hoechst 33342 staining (Figure 1(b)), especially in the high-dose group. These morphological observations indicated the concentration-dependent antitumor effects of BA on MM cells.

### 3.2. BA Inhibits MM Cell Viability and Proliferation

To objectively investigate the antitumor activities of BA against MM cells, we first employed the CCK-8 assay to evaluate cytotoxic effects. As shown in Figure 1(c), cell viability was inhibited in a concentration-dependent manner in both cell lines. Additionally, the EdU assay visually suggested the inhibitory effects of BA (Figure 1(d)). After treatment with different concentrations of BA for 12 h, the frequency of red-fluorescent MM cells (proliferative cells) was significantly decreased (Figure 1(e)). Thus, we confirmed that BA has a potent inhibitory effect on MM cells *in vitro*.

### 3.3. BA Activates the Mitochondrial Apoptosis Pathway in U266 Cells

**Figure 2:**
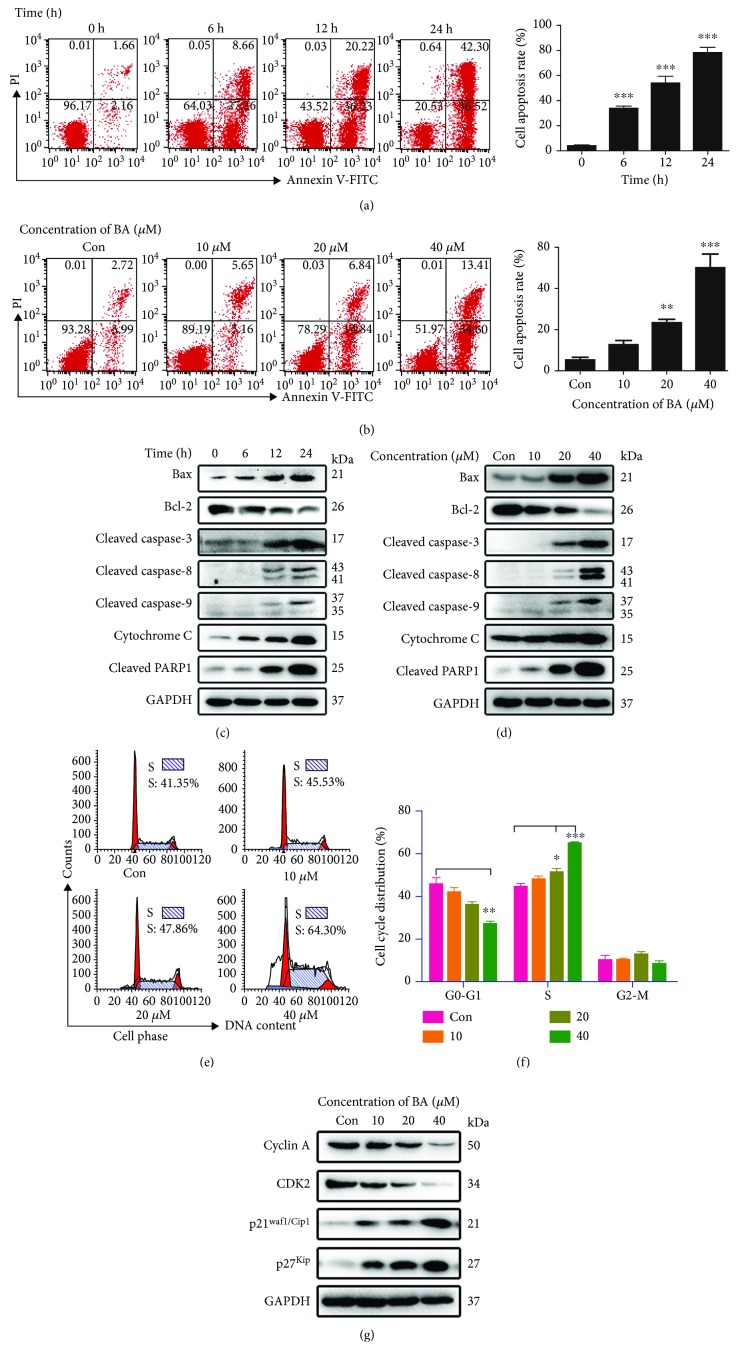
BA promoted apoptosis and S-phase arrest in U266 cells. (a) BA (40 *μ*M) was applied to U266 cells for different periods of time, and representative graphs and statistical analysis of annexin V/PI double staining are displayed; ^∗∗∗^*P* < 0.001. (b) U266 cells were cultured with the indicated concentrations of BA for 12 h, and representative flow cytometry graphs and statistical analysis of apoptosis are shown. ^∗∗^*P* < 0.01; ^∗∗∗^*P* < 0.001. (c, d) The expression levels of the mitochondrial apoptosis proteins Bax, Bcl-2, cleaved caspase-3, caspase-8, and caspase-9, cytochrome C, and cleaved PARP1 were evaluated by Western blotting after treatment with 40 *μ*M BA for the indicated times or after exposure to the indicated concentrations for 12 h. (e, f) The indicated concentrations of BA were applied to U266 cells for 12 h. Representative graphs and quantitative cell cycle analysis after flow cytometry. ^∗^*P* < 0.05; ^∗∗^*P* < 0.01; and ^∗∗∗^*P* < 0.001. (g) Expression levels of the S-phase-related proteins cyclin A, CDK2, p21^Waf1/Cip1^, and p27^Kip^ were detected by Western blotting.

We next measured cell apoptosis, which is a crucial process causing cell death. BA at 40 *μ*M increased the number of apoptotic cells in a time-dependent manner (Figure 2(a)). Similarly, increasing concentrations of BA resulted in higher proportions of apoptotic cells (Figure 2(b)). These results indicate that BA promotes apoptosis in a time- and concentration-dependent manner. To further investigate the potential proapoptotic molecules involved, mitochondrial apoptosis proteins were detected using Western blot. As shown in Figures 2(c) and 2(d), levels of the antiapoptotic effector Bcl-2 were decreased, whereas levels of the proapoptotic effector Bax increased in a time- and concentration-dependent manner. Moreover, we further confirmed that BA mediated the release of cyt c and activated cleaved caspase-3, caspase-8, and caspase-9 and cleaved PARP1 (Figures 2(c) and 2(d)), indicating that the mitochondrial apoptosis pathway was invoked in the process of the BA-mediated cell death.

### 3.4. BA Mediates S-Phase Arrest in U266 Cells

As another potent antitumor indicator, the cell cycle phase was examined in treated cells. Our representative flow cytometry plots (Figure 2(e)) and statistical analysis (Figure 2(f)) of U266 cells showed that BA exerted its antiproliferative effect by increasing the percentage of S-phase cells. However, no significant increase was observed at a low concentration level (Figure 2(f)), suggesting that other mechanisms of inducing cell death were functional at low concentrations. The proteins cyclin A, CDK2, p21^Waf1/Cip1^, and p27^Kip^ are important molecules for S-phase arrest [27], and as shown in Figure 2(g), BA concentration dependently decreased cyclin A and CDK2 but increased p21^Waf1/Cip1^ and p27^Kip^. These results further demonstrated the BA-induced S-phase arrest.

### 3.5. BA Causes MMP Collapse in U266 Cells

**Figure 3:**
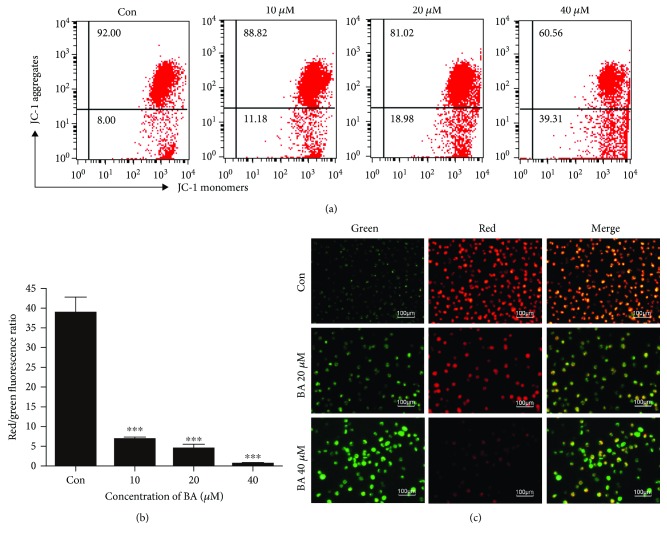
BA induced MMP loss in U266 cells. (a) Representative flow cytometry graphs of MMP after treatment with the indicated concentrations of BA for 12 h. (b) Quantitative analysis of the red-to-green fluorescence ratio; ^∗∗∗^*P* < 0.001. (c) Representative fluorescence graphs of MMP transition in U266 cells after JC-1 staining.

MMP is an important parameter of mitochondrial function, and MMP transition is generally perceived as an early sign of apoptosis. Flow cytometry plots (Figure 3(a)) and statistical analysis (Figure 3(b)) showed that BA induced a concentration-dependent decrease in red/green fluorescence ratios in U226 cells, as indicated by a shift from red JC-1 aggregates to green JC-1 monomers. In Figure 3(c), the control group mainly exhibited a higher incidence of red fluorescence, while the BA-treated group showed an obvious transition to green fluorescence, which indicated damaged mitochondria.

### 3.6. BA Induces ROS-Mediated U266 Cell Death

**Figure 4:**
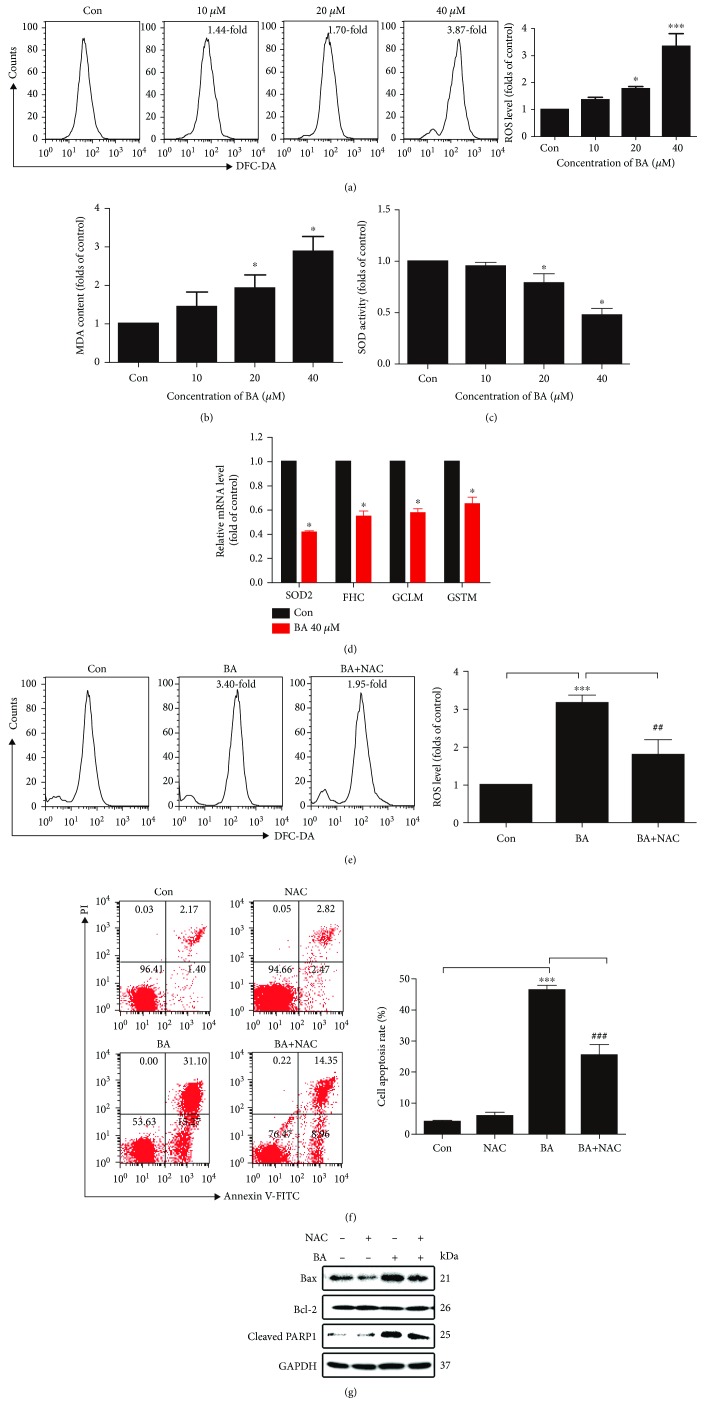
BA induced ROS accumulation in U266 cells in a concentration-dependent manner. (a) Representative plots of flow cytometric analysis using a DCFH-DA probe after 12 h of treatment and the quantitative analysis of the intracellular ROS level. ^∗^*P* < 0.05 and ^∗∗∗^*P* < 0.001. (b) Intracellular MDA content in different concentrations of BA; ^∗^*P* < 0.05. (c) SOD activity in different concentrations of BA; ^∗^*P* < 0.05. (d) Expression of SOD2, FHC, GCLM, and GSTM by real-time PCR after 40 *μ*M BA for 12 h; ^∗^*P* < 0.05. (e) Representative cytometric graphs and quantitative analysis of the ROS generation level with or without 10 mM NAC for 12 h. ^∗∗∗^*P* < 0.001*vs.* the control group; ^##^*P* < 0.01*vs.* the BA-treated group. (f) U266 cells were treated with 40 *μ*M BA with or without 10 mM NAC, the apoptosis rate was detected by flow cytometry, and quantitative analysis was shown. ^∗∗∗^*P* < 0.001*vs.* the control group; ^###^*P* < 0.001*vs.* the BA-treated group. (g) Expression levels of apoptosis-related proteins were evaluated by Western blotting after treatment with or without 10 mM NAC.

Mitochondria are important sources of intracellular ROS, and accumulation of ROS is closely related to cellular proliferation and apoptosis [12, 14]. After 12 h of treatment with BA (0, 10, 20, and 40 *μ*M), U266 cells exhibited concentration-dependent increases in intracellular ROS (Figure 4(a)). To further confirm the oxidative function of BA, oxidation product MDA and reductive substance SOD were measured. As shown in Figures 4(b) and 4(c), there is a concentration-dependent increase in MDA contents and reduction in SOD activities, especially for the high concentration group. There are many genes participating in the regulation of ROS. Moreover, our real-time PCR results showed that the expression of genes SOD2, FHC, GCLM, and GSTM was all decreased following treatment with BA (40 *μ*M) (Figure 4(d)), which indicate the underlying regulatory targets of ROS. When we investigated the role of ROS in BA-induced cell death, we firstly observed that BA-induced (40 *μ*M) ROS was significantly decreased by 10 mM NAC (Figure 4(e)). Subsequently, we detected apoptosis in the presence or absence of 10 mM NAC (Figure 4(f)). As expected, when ROS levels were reduced with the ROS scavenger NAC, apoptosis was effectively attenuated. In addition, the expression levels of Bax and cleaved PARP1 were decreased, while Bcl-2 levels were increased upon exposure to NAC (Figure 4(g)). Overall, our results suggest that BA exerted its apoptosis-promoting effect in U266 cells by enhancing intracellular ROS generation.

### 3.7. BA Blocks the NF-*κ*B Pathway to Promote ROS Accumulation in U266 Cells

**Figure 5:**
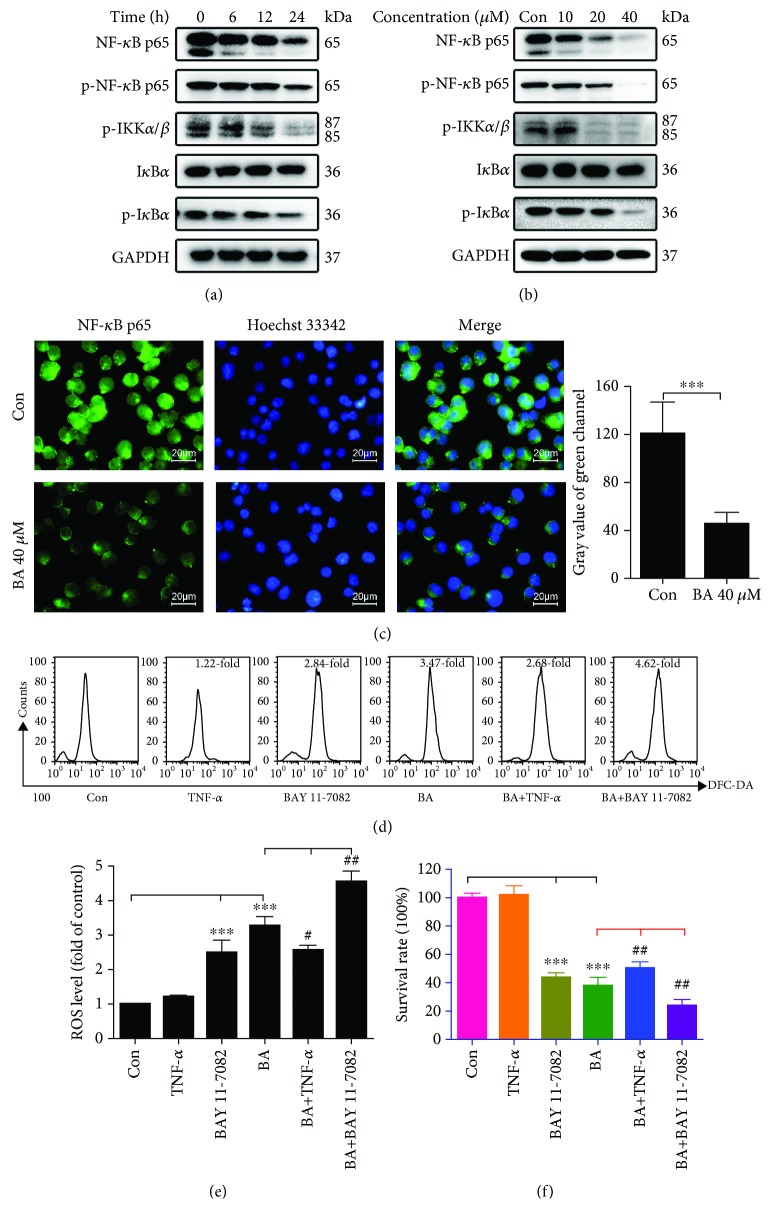
BA induced inhibition of the NF-*κ*B pathway, resulting in accumulation of ROS in U266 cells. (a, b) The expression levels of NF-*κ*B p65, p-NF-*κ*B p65, p-IKK*α*/*β*, I*κ*B*α*, and p-I*κ*B*α* in U266 cells were evaluated by Western blotting after exposure to 40 *μ*M BA for the indicated times or after exposure to the indicated concentrations for 12 h. (c) U266 cells incubated with 40 *μ*M BA or 0.1% DMSO for 12 h were analyzed for NF-*κ*B p65 expression and distribution by immunofluorescence; ^∗∗∗^*P* < 0.001. (d) Representative flow cytometry plots using DCFH-DA after treatment with 40 *μ*M BA for 12 h in the presence or absence of 10 ng/ml TNF-*α* and 5 *μ*M BAY 11-7082. (e) Quantitative analysis of ROS levels with or without TNF-*α* and BAY 11-7082 in BA-treated U266 cells. ^∗∗∗^*P* < 0.001*vs.* the control group; ^#^*P* < 0.05*vs.* the BA-treated group; and ^##^*P* < 0.01*vs.* the BA-treated group. (f) Quantitative analysis of survival rates by a CCK-8 assay via comparison between groups in the presence or absence of TNF-*α* and BAY 11-7082 and exposure to BA. ^∗∗∗^*P* < 0.001*vs.* the control group; ^##^*P* < 0.01*vs.* the BA-treated group.

We then determined whether BA has an effect on the NF-*κ*B pathway. According to the Western blot results, BA significantly reduced the levels of NF-*κ*B p65 and p-NF-*κ*B p65 in a time- and concentration-dependent manner (Figures 5(a) and 5(b)). Moreover, the immunofluorescence staining indicated lower expression and less nuclear binding levels of NF-*κ*B p65 (Figure 5(c)). The activated IKK complex is responsible for I*κ*B phosphorylation and degradation, thus contributing to the release and translocation of free NF-*κ*B dimers. The p-IKK*α*/*β* and p-I*κ*B*α* were all time- and concentration-dependently decreased after BA treatment (Figures 5(a) and 5(b)). The above results demonstrate that BA strongly inhibits the NF-*κ*B pathway.

Additionally, it has been reported that NF-*κ*B suppresses ROS levels in cancer cells [25]. To explore the regulatory relationship between these two factors, we chose the pathway-specific activator TNF-*α* and the inhibitor BAY 11-7082 to stimulate and suppress the NF-*κ*B pathway, respectively, and then assessed intracellular ROS levels and cell viability. Our results showed that the pathway-specific activator partially reversed the elevated ROS levels (Figures 5(d) and 5(e)) and cytotoxicity induced by BA (Figure 5(f)), whereas the pathway inhibitor acted in the opposite fashion. These findings indicate that blocking this pathway has the potential to elicit strong antitumor responses, partly through breaking cellular redox homeostasis.

### 3.8. BA Inhibits the Growth of MM Xenograft Tumors *In Vivo*

**Figure 6:**
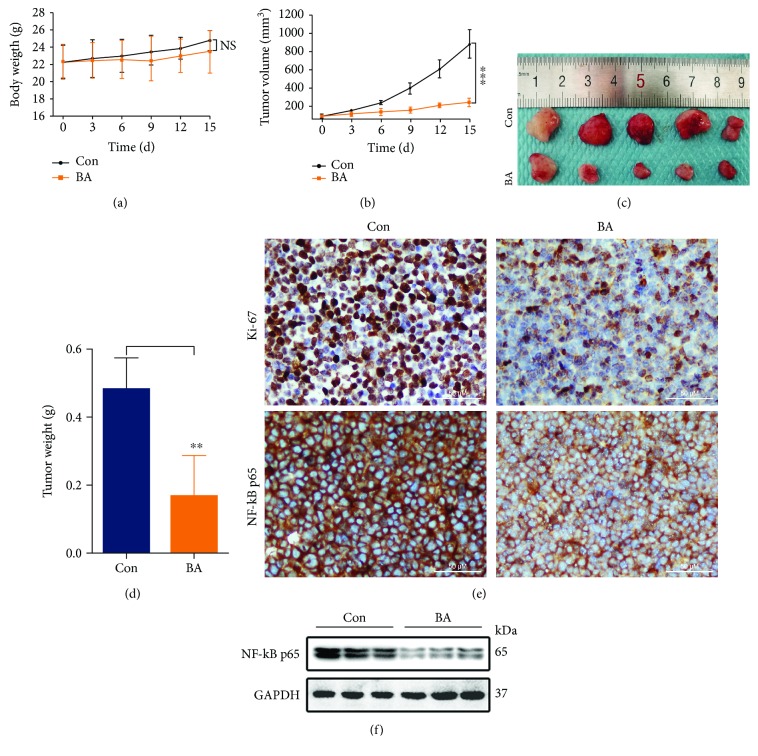
BA inhibited MM xenograft tumor growth *in vivo*. Tumor-bearing mice were randomly assigned to the control and BA-treated groups. The body weights (a) and tumor volumes (b) were measured every third day. NS indicates no significant difference *vs.* the control group; ^∗∗∗^*P* < 0.001. (c) Photographs of isolated tumors. (d) The weights of stripped tumors; ^∗∗^*P* < 0.01. (e) Tumor tissues were stained with Ki-67 and NF-*κ*B p65 via immunohistochemistry. (f) The level of NF-*κ*B p65 in xenograft tumors was assessed by Western blotting.

We further performed *in vivo* studies in nude mice. Indeed, chemotherapy is often accompanied by dramatic side effects, such as substantial body weight loss. Interestingly, no significant difference in body weight loss existed between the two groups (Figure 6(a)). As is statistically and visually displayed in Figures 6(b) and 6(c), the xenograft volumes in the BA-treated group were significantly reduced (inhibition ratio of approximately 72.1%). Moreover, the tumor weights (Figure 6(d)) of the experimental group (0.17 ± 0.05 g) were much lower than those of the control group (0.48 ± 0.04 g). Assessment of cellular proliferation by Ki-67 immunohistochemistry is a consistent and powerful prognosticator in MM [28]. Our IHC graphs showed that proliferating cells with brown-stained Ki-67^+^ nuclei were more frequently observed in the control group (Figure 6(e)). Consistent with our *in vitro* experiments, BA inhibited the expression of NF-*κ*B p65 *in vivo*, as indicated by IHC and Western blot results (Figures 6(e) and 6(f)).

## 4. Discussion

Studies explosively report the treatments currently in use for MM. These therapies include high-dose chemotherapy, autologous stem cell transplant, cell immunotherapy, and novel agents [2]. However, few studies have explored traditional Chinese medicines, such as BA. Herein, we focus on the anticancer activities of BA against MM *in vitro* and *in vivo*. We demonstrate that BA induces apoptosis, S-phase arrest, MMP collapse, and ROS overproduction. Subsequently, we investigate the effects of BA on NF-*κ*B signaling and finally reveal the role of the NF-*κ*B system on ROS. To our knowledge, our study is the first to report that BA inhibits MM cells by suppressing NF-*κ*B transcriptional activity, which in return disrupts redox homeostasis.

The mitochondrial pathway is believed to be one of the most crucial mechanisms of BA-mediated cell death in various cancers [9, 29, 30]. Molecularly, Bcl-2 families, regulating the balance of proapoptotic (Bax) and antiapoptotic (Bcl-2) proteins, determine whether a cell survives or undergoes apoptosis [31]. As systematically summarized, the mitochondrial apoptosis pathway is activated by a litany of stimuli that trigger the release of cyt c, facilitate the formation of the apoptosome, and activate apical caspases (caspase-8 and caspase-9) and then downstream caspase-3, which results in cell death [12, 32]. Additionally, we revealed that BA effectively activated apoptosis in a process dependent on the upregulation of Bax, the downregulation of Bcl-2, and the activation of cleaved caspase-3, caspase-8, and caspase-9, cyt c, and cleaved PARP1. All of these evidences indicate that the mitochondrial apoptosis pathway plays a crucial role in BA-mediated U266 cell death.

Loss of cell checkpoint controls is one of the hallmarks of tumorigenesis. Treatments targeting the cell cycle are emerging as a most promising cancer therapy, and intense efforts are now underway [27]. Goswami et al. showed that BA induced G_0_/G_1_ arrest in SiHa cells [15]. However, it was reported that BA blocked breast cancer at the G_2_/M checkpoint [33], whereas its derivatives mainly targeted the S-phase in human hepatocellular carcinoma and leukemia [34]. Such cell-selective and tumor-specific characteristics indicate that a certain checkpoint block may occur in BA-treated U266 cells. Our findings confirmed that BA-treated MM stalled at the S-phase. Molecularly, the cell cycle, which involves a series of tightly integrated events, is regulated by cyclin/CDK complexes, which are again modulated by a host of CDK inhibitors [27]. We confirmed that S-phase arrest was mainly driven by a synergistic mechanism involving inhibition of the cyclin A2/CDK2 complex as well as stimulation of p21^Waf1/Cip1^ and p27^Kip^.

In comparison to their normal counterparts, cancer cells inherently obtain a moderately elevated ROS level, which contributes to cancer initiation, malignancy, and resistance to chemotherapy [14]. Nevertheless, the elevated ROS usually plays a deleterious role [13]. BA clearly induces overwhelming oxidative stress, which initiates cell death in many cancers [15, 16, 30]. Similar results were observed in our study, that is, BA concentration-dependently induced depolarization of MMP, accumulation of ROS, and activation of the mitochondrial pathway. These findings suggest that ROS mediate a unique way of eliminating MM cells, as further confirmed by our results that cell death was partially attenuated by ROS scavengers (Figure 4).

The NF-*κ*B system, which regulates the tumor microenvironment and modulates inflammation, tumorigenesis, and therapy resistance [10, 17, 20], has long been proven to be implicated in MM [35]. However, the BA-mediated transcriptional activities of NF-*κ*B have caused massive controversies, namely, BA blocks NF-*κ*B activity to inhibit cervical cancer [36], prostate cancer [26], and breast cancer [19]. Whereas, in human endometrial adenocarcinoma [37] and prostate cancer of LNCaP cells [23], BA activates the NF-*κ*B pathway to sensitize cells to death. Thus, we sought to determine the transcriptional levels of the NF-*κ*B pathway in BA-treated U266 cells. In accordance with the majority of findings, our results showed that BA may act as a potent NF-*κ*B pathway inhibitor, not an activator.

The NF-*κ*B pathway also closely interacts with ROS. Researchers have reported that upregulated NF-*κ*B activities promote expression of downstream antioxidant genes, such as the ROS-related genes ferritin heavy chain (FHC) and SOD2 [24], thereby protecting cells from the insults of oxidative damage. Moreover, our results found that upregulated NF-*κ*B activity partially reversed the excess ROS and attenuated cytotoxicity induced by BA, whereas BA and the pathway-specific inhibitor BAY 11-7082 exerted synergistic cytotoxic effects by further elevating ROS. Therefore, we conclude that the inhibition of the NF-*κ*B pathway enhances downstream oxidant effectors and promotes ROS production in BA-treated MM cells.

## 5. Conclusion

**Figure 7:**
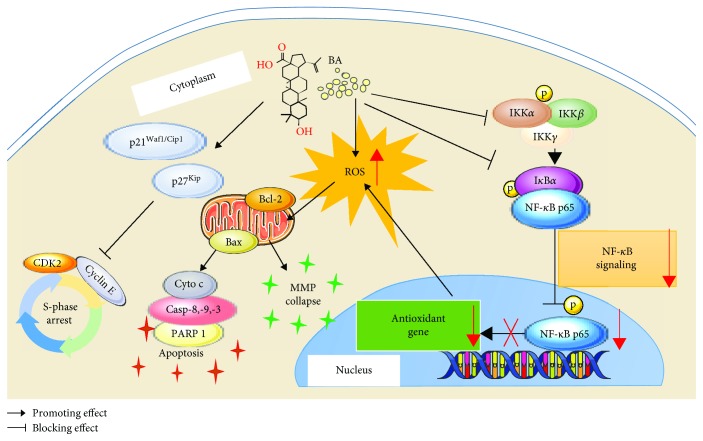
Illustration of the potential mechanisms occurring in BA-treated MM cells.

In our novel findings, we intensively elaborated on the inhibitory effects of BA on MM cells both *in vitro* and *in vivo*. The potential mechanisms mainly include mitochondrial apoptosis induction, cell cycle blockade, MMP disruption, intracellular ROS accumulation, and NF-*κ*B signaling inhibition (Figure 7). And we especially elucidated the complex regulatory roles between ROS and the NF-*κ*B pathway in MM. Overall, BA provides valuable insight for clinical applications in treating MM, though further studies are warranted to reveal its broader mechanisms and to ensure its drug safety.

## Data Availability

The data used to support the findings of this study are available from the corresponding author upon request.
